# Molecular evidence of upper and lower respiratory infection due to *Lophomonas* in a post‐kidney transplantation patient

**DOI:** 10.1002/ccr3.5492

**Published:** 2022-02-20

**Authors:** Mahdi Fakhar, Sepideh Safanavaei, Maryam Nakhaei, Samira Esmaeili, Elham Sadat Banimostafavi, Fatemeh Spahbodi, Ali Sharifpour

**Affiliations:** ^1^ Toxoplasmosis Research Center Communicable Diseases Institute Iranian National Registry Center for Lophomoniasis (INRCL) Imam Khomeini Hospital Mazandaran University of Medical Sciences Sari Iran; ^2^ Pulmonary and Critical Care Division Imam Khomeini Hospital Mazandaran University of Medical Sciences Sari Iran; ^3^ Department of Radiology Imam Khomeini Hospital Mazandaran University of Medical Sciences Sari Iran; ^4^ Department of Nephrology Imam Khomeini Hospital Mazandaran University of Medical Sciences Sari Iran

**Keywords:** kidney transplantation, *Lophomonas*, sinusitis, tonsillitis

## Abstract

We report a case of lophomoniasis in a kidney post‐transplantation patient. The patient, 46‐year‐old man, had pneumonia, acute sinusitis, and tonsillitis on admission. We recommend that lophomoniasis should be essentially ruled out in all patients suffering from post‐transplantation infection, particularly in those who do not respond to routine antibiotic regimens.

## Graphical abstract

Lophomoniais in patients with a history of kidney post‐transplant infection who have symptoms of acute sinusitis and tonsillitis that do not respond to standard antibiotic regimens should be screened.

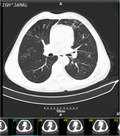

## 1 INTRODUCTION

Over the last decade, the incidence of lophomoniasis, caused by *Lophomonas blattarum* (*L*.* blattarum*), has been increased mostly in Asian countries such as Iran and China.^1^, ^2^, ^3^, ^4^, ^5^ This emerging protozoan parasite can cause upper and lower respiratory infections, mainly pulmonary lophomoniasis (PL), with nonspecific symptoms such as prolonged cough, dyspnea, low‐grade fever, and broncho‐pulmonary abnormalities.^2^, ^4^, ^5^ It is commonly found in the hindgut of cockroaches, and humans can be infected via inhalation of the infective cyst.^5^ Currently, microscopic examination of bronchoalveolar lavage (BAL) fluid, bronchial aspirate, and sputum samples is a common method for the diagnosis of lophomoniasis.^1^, ^4^, ^5^ However, a PCR test for identifying the parasite in various clinical samples has recently been developed to avoid misdiagnosis in this regard.^6^

Serious complications due to opportunistic pathogens following kidney transplantation are still one of the leading causes of death in the post‐transplant period.^7^ Thus, the satisfactory outcome of renal transplants depends on the early diagnosis and suitable treatment of infectious complications.^7^ Organ transplant recipients are particularly susceptible to opportunistic infections because of the continuous suppression of their immune systems. Several pathogens such as viral, fungal, and parasitic agents have been reported in post‐transplant infection patients. In this regard, lung infections in renal transplantation patients in the early and late post‐transplant period have been reported.^7^, ^8^, ^9^, ^10^

There is, however, some evidence confirming that *Lophomonas* can infect immunocompromised patients, including organ transplant recipients.^11^, ^12^ However, there are several cases of lophomoniasis that have been reported in immunocompetent patients.^1^, ^2^, ^3^, ^6^ To date, there is no report regarding co‐existence of pulmonary lophomoniasis and tonsils and paranasal sinuses involvement simultaneously in immunocompromised and or immunocompetent patients worldwide. Thus, the clinical manifestations, history, and molecular detection of late‐onset *Lophomonas* infection in a patient with kidney transplantation referred to the Iranian National Registry Center for Lophomoniasis (INRCL) at Mazandaran University of Medical Sciences, Imam Khomeini teaching hospital, Sari, northern Iran, are described here.

## 2 CASE STUDY

A 46‐year‐old man, a non‐smoker farmer, was admitted to the Department of Respiratory Medicine at Imam Khomeini Hospital at Mazandaran University of Medical Sciences, Sari, in September 2018 due to a prolonged mild fever, cough, and hemoptysis. Symptoms had begun a month before the recent admission and were characterized by low‐grade fever and productive coughing with green sputum and occasional bloodshed with blood or clear blood of less than 1 degree, which was why the patient was admitted and, after receiving venous antibiotics for 1 week, they were discontinued, but 15 days later, they were reintroduced due to the lack of symptoms.

The patient had a kidney transplant 10 years ago and had no acute problem in this regard, but during these 10 years, it was regularly recurring due to recurrent tonsillitis and repeated symptoms, but despite repeated antibiotics, he did not improve. A year ago, after traveling to Iraq, he had symptoms of pneumonia and hemoptysis and had been hospitalized four times in a year.

**FIGURE 1:**
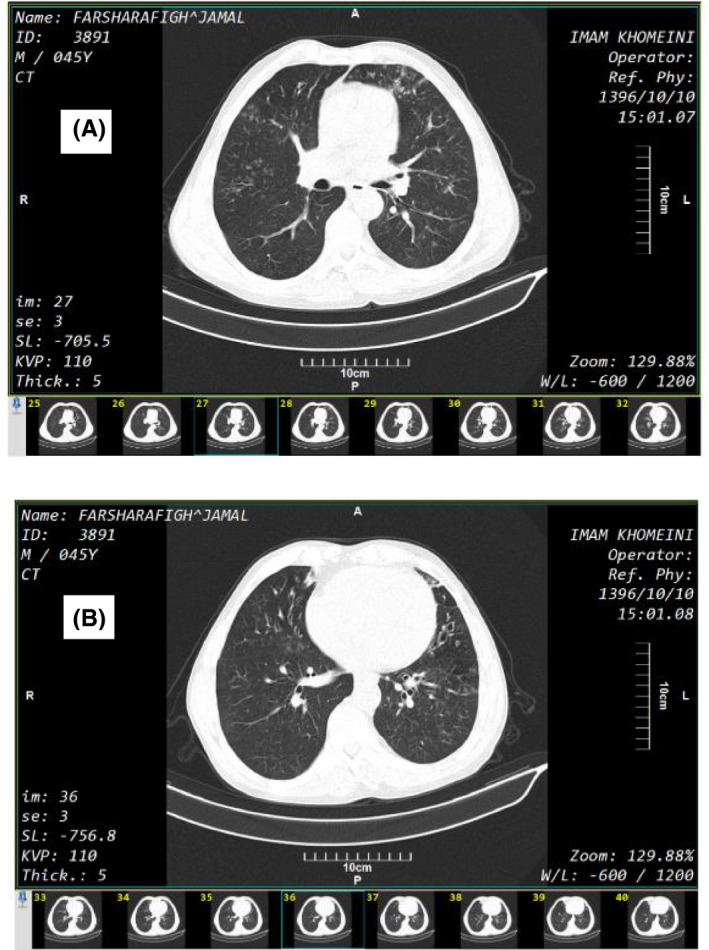
Lung computed tomography (CT) scan of the patient with *Lophomonas* infection showing scattered micronodules with tree in bud pattern (A) and associated bronchiectasis in lingula (B)

His vital signs were as follows: blood pressure (systolic/diastolic):140/80 mmHg, body temperature: 38.5°C oral, saturated pressure O2 (SPO2): 95 air room, respiratory rate: 24. In physical examination, the patient was not ill and toxic. He had no breath distress and tonsils were enlarged and erythematous in cryptogenic position. Lung computed tomography (CT) scans showed nonspecific radiological changes including scattered micronodules with tree in bud pattern (A) and associated bronchiectasis in lingula (see Figure 1).

His routine laboratory biochemical tests and complete blood count (CBC) were as follow: WBC:14000, Poly Morpho Nuclear (PMN):80%, Lym:18%, Hb: 10 mg/dl, MCV:85 fl, Plt: 400000, Creatinine (CR):1/9, CR:1/7, Blood Urea Nitrogen (BUN):14, The Erythrocyte Sedimentation Rate (ESR) 65 mm/h, C‐Reactive Protein (CRP): 2 plus. The sputum smear was negative for *Mycobacterium tuberculosis*. On the fourth day of hospitalization, a diagnostic bronchoscopy was performed and the two BAL and sputum samples were sent to the pathology laboratory and INRC for rule out of the microbial pathogens, cytology examination, and lophomoniasis, respectively. The laboratory findings were as follows: in a wet smear prepared from the BAL fluid sample, the motile multi‐flagellated *Lophomonas* was detected under a light microscope. As well as, the parasite was found in a smear prepared from sputum specimen. Moreover, a 214 bp band corresponding to *Lophomonas* spp., using genus‐specific PCRwas observed. Thus, the infection was also confirmed by a molecular evidence.^5^

His drug history was Mycophenolate Mofetil (Cell cept) 500 mg/BD (Bis in Die), Prednisolon 5 mg/daily, Sandimun 50 mg/BD, Alprazolam 0.5 mg/daily, and Fluvoxamine 50/daily. In hospital, meropenem and ciprofloxacin were prescribed for the patient. Additionally, metronidazole ampoule 500 mg TDS (Ter Die Sumendum) was added to previous antibiotics. Patient's fever and hemoptysis were stopped after 48 hours. After 6 days, the patient was given a good general condition and metronidazole (500 mg of TDS, orally) was ordered for 8 weeks.

Two months later, the symptoms of tonsillitis and post‐nasal discharge (PND) were significantly improved, and the pulmonary symptoms were significantly reduced. The patient was again treated for metronidazole 500 mg TDS, orally, for 2 weeks to avoid relapse. On 6‐month follow‐up, the patient was cured completely and had no previous symptoms.

## 3 DISCUSSION

Infectious agents are the second most common cause of death among in the early post‐transplant period. Frequently, 2–6 months after transplantation, due to the high status of immune suppression, infectious agents, like fungal and parasitic agents, especially opportunistic ones, will invade various organs of the body, including the lungs.^7^, ^8^, ^9^

Lophomoniasis has been reported in patients with immunosuppression, such as solid and non‐solid organ transplant recipients,^11^, ^12^, ^13^ as well as in immunocompetent patients with conditions such as asthma,^1^ diabetes mellitus,^1^ sinusitis,^6^ and acute myeloid leukemia (AML‐2).^14^ Moreover, recently co‐morbidity of emerged *Lophomonas* infection and COVID‐19, pulmonary aspergillosis and tuberculosis have been reported from Iran.^1^, ^15^, ^16^ However, our patient with renal transplantation history was suffering concurrently from upper and lower respiratory involvement due to a *Lophomonas* infection for a long time. To the best of our knowledge, this is the first report regarding simultaneous pneumonia, tonsillitis, and acute sinusitis due to *Lophomonas* infection.

In general, this is due to the fact that the clinical signs and symptoms of the patient with lophomoniasis and even radiological findings are completely nonspecific and are indistinguishable from other infections or similar diseases.^1^, ^16^, ^17^ The main characteristics of pulmonary lophomoniasis are a persistent cough, dyspnea, and radiological abnormalities.^4^, ^5^, ^11^, ^17^ These findings are absent in the early stages of *Lophomonas* infection, which is in contrast to tuberculosis, pulmonary *Strongyloides stercoralis* infection, *Pneumocystis jiroveci* pneumonia (PCP), cytomegalovirus (CMV) pneumonia, and tuberculosis.^7^, ^8^, ^9^, ^10^

In the present study, *Lophomonas* was found both in BAL fluid and sputum specimens, which demonstrates that it could infect both the lower and upper parts of the pulmonary system. Accordingly, the diagnosis of *Lophomonas* infection based on clinical and radiological findings is not possible alone. At present, diagnosis of lophomoniasis is mainly based on microscopic observation of the parasite in a wet mount and or following a staining technique such as Papanicolaou and Giemsa stains under a light microscope.^1^, ^4^, ^15^, ^16^, ^18^, ^19^ The disadvantages of this method include misdiagnosis of the disease due to the similarity of the parasite to atypical and or degenerated bronchial cells, such as ciliocytophthoria, and its low sensitivity due to small amounts of the clinical sample.^1^, ^19^ Therefore, microscopic examination of the disease requires a lot of experience and appropriate amounts of the sample. On the contrary, due to recent advances in the molecular diagnosis of this infection, it seems that in cases of doubt, the use of PCR test is strongly recommended to achieve a reliable diagnosis.^1^, ^6^ As a whole, if such infection is suspected, especially in post‐transplant conditions, in order to prevent possible complications and manage the patient's treatment, diagnostic bronchoscopy and examination of the BAL and or sputum specimens with accurate diagnostic methods such as specific PCR is recommended.

## 4 CONCLUSION

We recommend that lophomoniasis should be essentially ruled out in all patients suffering from post‐transplantation upper and lower respiratory system infection, particularly in those who do not respond to routine antibiotic regimens and with nonspecific radiological changes. However, in post‐transplantation patients who are resistant to antimicrobial treatments and with similar clinical findings, early work up to diagnose lophomonias is highly recommended. Even if, there was no access to a diagnostic laboratory test, empirical prescription of metronidazole could be useful to avoid some complications.

## CONFLICT OF INTEREST

The authors confirm that this article content has no conflict of interest.

## AUTHOR CONTRIBUTIONS

SS and MF involved in the interpretation and collecting of data and editing of the manuscript. SE and MN involved in writing, editing, and preparing the final version of the manuscript. MF and Ash, ESB, and FS involved in critically revising the whole manuscript. All authors reviewed the paper and approved the final version of the manuscript.

## ETHICAL APPROVAL

This research was approved by the research ethics committee of Mazandaran University of Medical Sciences (IR.MAZUMS.REC.1397.2969). Written informed consent was taken from the patient to include the clinical details.

## CONSENT

Informed consent for publication of this case report was taken from the patient.

## Data Availability

The data are available with the correspondence author and can be reached on request.
